# Development of Human Somatosensory Cortical Functions – What have We Learned from Magnetoencephalography: A Review

**DOI:** 10.3389/fnhum.2014.00158

**Published:** 2014-03-17

**Authors:** Päivi Nevalainen, Leena Lauronen, Elina Pihko

**Affiliations:** ^1^BioMag Laboratory, Hospital District of Helsinki and Uusimaa, HUS Medical Imaging Center, Helsinki University Central Hospital, University of Helsinki, Helsinki, Finland; ^2^Department of Clinical Neurophysiology, Children’s Hospital, HUS Medical Imaging Center, Helsinki University Central Hospital, University of Helsinki, Helsinki, Finland; ^3^Brain Research Unit, O.V. Lounasmaa Laboratory, Aalto University School of Science, Espoo, Finland

**Keywords:** magnetoencephalography, newborn, brain development, somatosensory system, preterm infant, cerebral palsy

## Abstract

The mysteries of early development of cortical processing in humans have started to unravel with the help of new non-invasive brain research tools like multichannel magnetoencephalography (MEG). In this review, we evaluate, within a wider neuroscientific and clinical context, the value of MEG in studying normal and disturbed functional development of the human somatosensory system. The combination of excellent temporal resolution and good localization accuracy provided by MEG has, in the case of somatosensory studies, enabled the differentiation of activation patterns from the newborn’s primary (SI) and secondary somatosensory (SII) areas. Furthermore, MEG has shown that the functioning of both SI and SII in newborns has particular immature features in comparison with adults. In extremely preterm infants, the neonatal MEG response from SII also seems to potentially predict developmental outcome: those lacking SII responses at term show worse motor performance at age 2 years than those with normal SII responses at term. In older children with unilateral early brain lesions, bilateral alterations in somatosensory cortical activation detected in MEG imply that the impact of a localized insult may have an unexpectedly wide effect on cortical somatosensory networks. The achievements over the last decade show that MEG provides a unique approach for studying the development of the somatosensory system and its disturbances in childhood. MEG well complements other neuroimaging methods in studies of cortical processes in the developing brain.

## Introduction

**Figure 1:**
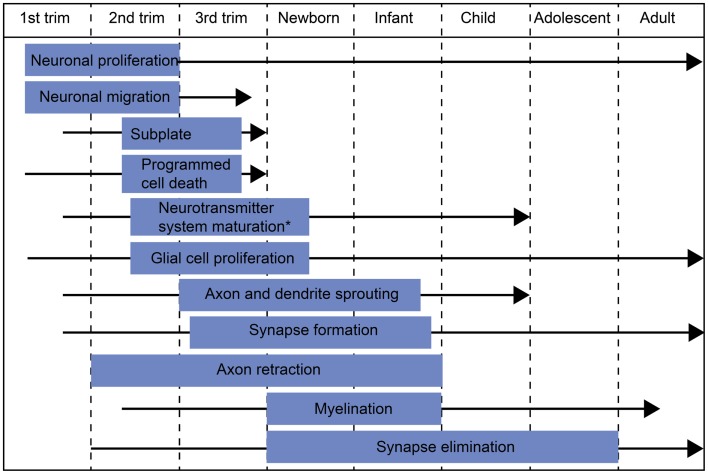
**Schematic diagram of major developmental events during embryonic/fetal life and infancy/early childhood**. The blue box indicates the most active period of each developmental process and the black arrow the period when the process continues at a slower pace. *The most active period in neurotransmitter system maturation in general. The exact timescales of maturation for different neurotransmitter systems may differ from the indicated period.

Around the time of full-term birth, the central nervous system (CNS) of a human newborn is developing dramatically (Figure 1). Transient fetal brain structures, such as the subplate zone, are resolving (Kostovic and Rakic, 1990; De Graaf-Peters and Hadders-Algra, 2006) and neurotransmitter systems are undergoing marked changes (Ben-Ari et al., 2004; Herlenius and Lagercrantz, 2004; Dzhala et al., 2005). The active phase in dendritic development and synaptogenesis continues for months to years after birth (Huttenlocher and Dabholkar, 1997), whereas myelination, axonal withdrawal, and synapse elimination may continue up to the third decade of life (Huttenlocher and Dabholkar, 1997). (For a review on the ontogeny of the human CNS, see De Graaf-Peters and Hadders-Algra, 2006.) Considering all of these ongoing changes, early infancy is a very exiting period to investigate the building of neural networks and their functional development.

In recent years, several non-invasive brain research methods have been introduced for *in vivo* studies of the developing CNS. Advanced magnetic resonance imaging (MRI) techniques [such as voxel-based morphometry and diffusion tensor imaging (DTI)] allow not only the visualization but also the quantification of gray and white matter structures (e.g., Mathur et al., 2010). Furthermore, functional MRI (fMRI) detects hemodynamic changes related to neural activation providing spatially accurate information about brain activation in response to a stimulus (for a review, see e.g., Seghier and Hüppi, 2010) or about the so called resting-state networks (for a review, see e.g., Smyser et al., 2011). Of the available neurophysiological methods, electroencephalography (EEG) and evoked potentials have a long history in studies of all age groups. Magnetoencephalography (MEG), on the other hand, has been used in studies of newborns and infants only relatively recently (for a review, see e.g., Huotilainen, 2006; Lauronen et al., 2011). All of these brain research methodologies have their pros and cons, and combining the results obtained with different methods provides a comprehensive picture of brain development. This review discusses the discoveries made with MEG concerning normal and abnormal development of the human somatosensory system in infancy and childhood.

Magnetoencephalography detects the weak extracranial magnetic fields produced by synchronous activity of tens of thousands of cortical pyramidal neurons. More specifically, the MEG signal is thought to reflect synaptically induced intracellular currents flowing in the apical dendrites of cortical pyramidal cells (Hämäläinen et al., 1993). Thus, similar to EEG, the temporal resolution of MEG is in the millisecond range. In the spatial domain, source localization is simpler for MEG than EEG data due to the inherently different properties of the two methods: MEG is less sensitive to conductivity differences between the measuring device and the active brain source, and MEG preferentially detects sources oriented tangentially to the skull surface, whereas EEG detects both radial and tangential sources (Hämäläinen et al., 1993). Consequently, with MEG, brain processes can be studied relatively accurately both in time and space.

**Figure 2:**
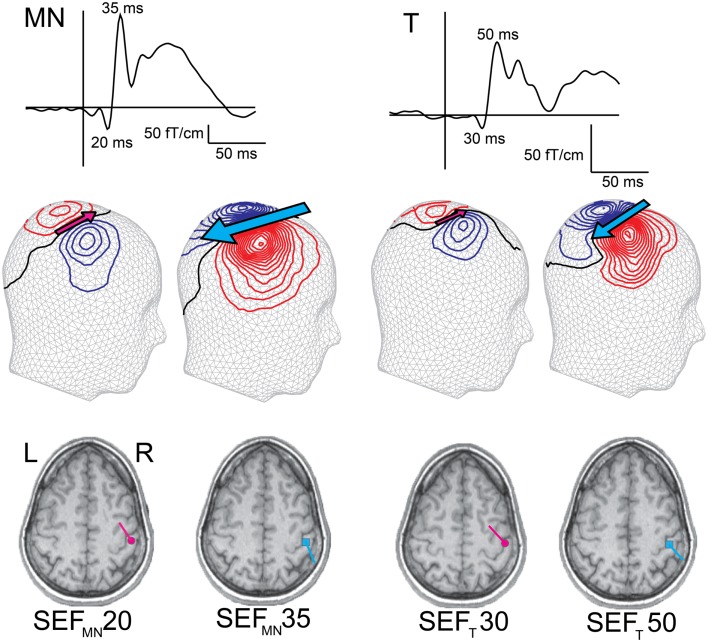
**Early adult SEFs to median nerve (SEF_MN_) and tactile (SEF_T_) stimulation**. The SEF waveform from one gradiometer channel shows SEF_MN_20/SEF_T_30 and SEF_MN_35/SEF_T_50 deflections, and the magnetic contours – reflected on a head surface – reveal the different current orientations (indicated by the pink and blue arrows). Compared with SEF_MN_, the SEF_T_ latencies are slightly longer and amplitudes lower (note the different amplitude scales). Both stimulation methods, however, elicit an initial anteriorly pointing dipolar source, SEF_MN_20/SEF_T_30 (though in some subjects this deflection is minute to tactile stimulation), followed by a posteriorly pointing dipolar source, SEF_MN_35/SEF_T_50. The dipoles are superimposed on individual MRIs and both sources are localized in SI.

Somatosensory responses can be evoked by electrical stimulation of a peripheral nerve (e.g., median nerve) or by tactile stimulation of the skin (e.g., on the digits). Stimulation of the median nerve at the wrist activates a mixture of afferent and efferent fibers, including those innervating many types of cutaneous receptors in about two thirds of the palmar side of the hand. In most of the experiments reviewed here, the tactile stimulation was provided with an inflatable plastic diaphragm driven with pulses of compressed air. Such a stimulus feels like a gentle tap on the fingertip and activates mainly slowly adapting mechanoreceptors in a relatively localized skin area. Compared with median nerve stimulation, the early somatosensory evoked field (SEF) deflections to tactile stimulation have usually lower response amplitudes, and slightly longer latencies (Figure 2), partly due to the more distal stimulation site (e.g., wrist vs. fingertip). Nevertheless, in adults, the early cortical SEFs to both median nerve (SEF_MN_) and tactile stimulation (SEF_T_) consist of an initial deflection with an underlying current source pointing anteriorly (though this deflection is often minute after tactile stimulation, Figure 2, SEF_MN_20 and SEF_T_30^1^), and a subsequent deflection with current pointing posteriorly (Figure 2, SEF_MN_35 and SEF_T_50). The following sections discuss how and when such somatosensory response patterns are attained in infancy and what kind of underlying processes this development might reflect.

## Normal Development of the Somatosensory System

In the following sections, we first review different aspects of somatosensory development and then discuss the relevant findings of developmental somatosensory MEG studies.

### From the periphery to the primary somatosensory areas

Postmortem studies in human infants have shown that in the primary somatosensory areas, thalamic axons grow through the subplate, a transient fetal structure underneath the cortical plate, between the 17 and 26th gestational weeks (GW^2^) (Kostovic and Rakic, 1990; Kostovic et al., 1995). During the early preterm period (26th–34th GW), these axons grow to the cortical plate and form the first thalamo-cortical connections, constituting the anatomical pathway for sensory impulses from the periphery to the cortex. Myelination starts in the human telencephalon around the 14th GW (Zecevic et al., 1998). In the pre- and post-central gyri, myelin is detectable around the 35th GW (Iai et al., 1997). Myelination then proceeds actively during the first postnatal year (Brody et al., 1987) and continues at a slower pace thereafter. In accordance with changes caused by pre-myelination and myelination, *in vivo* DTI data show a basic pattern of white matter maturation both before and after term-age. As a function of gestational age, mean diffusivity decreases and fractional anisotropy (a measure of relative degree of directionality of diffusion in a voxel) increases in a posterior-to-anterior and a central-to-peripheral order (Hüppi et al., 1998a; Berman et al., 2005; Yoshida et al., 2013).

In human infants, the functionality of the early connections from the periphery through thalamus to the primary somatosensory cortex (SI) can be explored *in vivo* with somatosensory evoked potentials (SEPs) recorded on the scalp, or with SEFs recorded extracranially with MEG. In preterm infants, median nerve SEPs are recordable on the scalp already by the 25th GW (Hrbek et al., 1973). In the youngest preterm infants (<30 GW), the most striking feature of the scalp SEP is a large negative wave with a mean duration of 1500 ms (Hrbek et al., 1973; Vanhatalo et al., 2009). This slow wave can be detected without averaging when a tactile stimulus is given between bursts of the *tracé discontinue* EEG pattern of preterm infants (Milh et al., 2007; Vanhatalo et al., 2009). A concerted action of the subplate and cortex may be required for generation of this component (Kanold, 2009; Vanhatalo et al., 2009). With increasing gestational age, the amplitude of this slow wave gradually decreases and an earlier component, usually referred to as N1 in the literature, becomes detectable with a latency of approximately 90 ms somewhere between the 27th (Taylor et al., 1996) and 29th GW (Hrbek et al., 1973). Toward term-age, the N1 latency rapidly decreases (Hrbek et al., 1973; Klimach and Cooke, 1988a; Karniski et al., 1992; Taylor et al., 1996; Smit et al., 2000), reaching approximately 30 ms at term-age (sep_MN_30), though with considerable inter-individual variability (Desmedt and Manil, 1970; Hrbek et al., 1973; Laget et al., 1976; Zhu et al., 1987; Laureau et al., 1988; Laureau and Marlot, 1990; George and Taylor, 1991; Gibson et al., 1992; Karniski, 1992).

In term-age infants, primary somatosensory responses have also been studied with MEG. In accordance with the neonatal sep_MN_30, the initial SEF to median nerve stimulation in full-term newborns peaks at around 30 ms (sef_MN_30). The MEG data together with earlier EEG findings suggest that this earliest cortical response in newborns reflects activation of a similar cortical ensemble as the earliest SEF_MN_ response in adults (SEF_MN_20 peaking at 20 ms) (Lauronen et al., 2006) – that is, summated intracellular currents in cortical pyramidal cells in SI, specifically area 3b in the anterior wall of postcentral gyrus (Allison et al., 1989a). Since the current direction in SI pyramidal cells during this early response is oriented from deeper to more superficial cortical layers, MEG source modeling yields an anteriorly pointing current dipole, which in SEP is recorded as a posterior negativity (SEP_MN_20 in adults and sep_MN_30 in infants).

**Figure 3:**
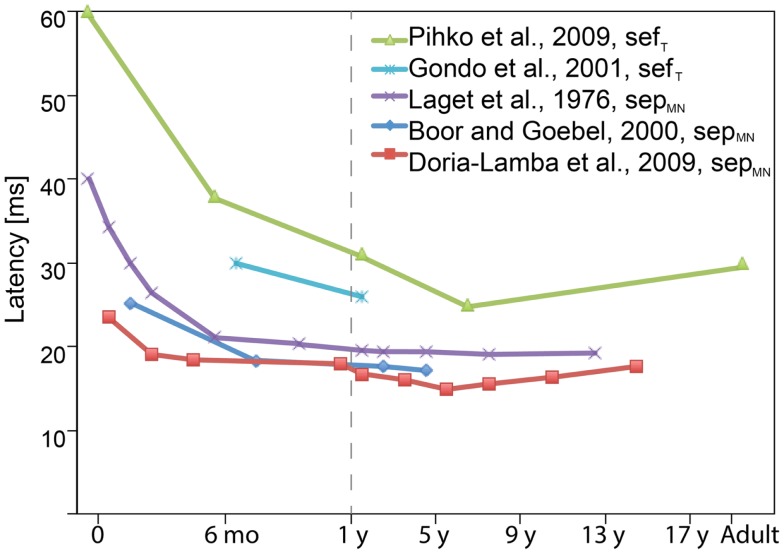
**Schematic diagram showing the latency evolution of the earliest cortical sep_MN_30 and sef_T_30 components with age as reported in various studies**. The general rule is that the sep_MN_30 and sef_T_30 latency decreases until the age of approximately 3–5 years, and slightly increases thereafter. The absolute latencies vary between studies due to differences in, e.g., stimulation methods and filter settings. Many of the studies also grouped subjects of different ages together in which case the figure displays the midpoint of the reported age range (when the average age was not reported). (Note the different age scales before and after 1 year.)

The development of this early response during childhood is primarily reflected in its latency (Figure 3). Although the absolute latencies differ between studies (due to, e.g., different stimulation method, filter settings, vigilance state/anesthesia), the general rule is that until the age of approximately 3–5 years, the sep_MN_30 and sef_T_30 latency decreases and slightly increases thereafter (e.g., Laget et al., 1976; Lauronen et al., 1997; Boor and Goebel, 2000; Gondo et al., 2001; Bercovici et al., 2008; Doria-Lamba et al., 2009; Pihko et al., 2009). These latency changes reflect the increasing neural conduction velocities following myelination and maturation opposed by physical growth of the body and limbs (Müller et al., 1994; Boor and Goebel, 2000; García et al., 2000).

### Development of local cortical circuits and intracortical processing

Besides the wiring of thalamo-cortical connections, major developmental changes also occur within the cortex during the second and third trimester as well as postnatally (Marin-Padilla, 1970). Between the 28 and 40th GW, the brain volume more than doubles and cortical gray matter volume extends fourfold (Hüppi et al., 1998b). Postmortem data of human infants show that development of dendrites of cortical pyramidal cells begins during the second trimester from the deeper cortical layers, followed by more superficial layers (Mrzljak et al., 1992). The number of basal dendrites stabilizes around the 27th GW, but their growth in length accelerates during the third trimester and continues postnatally (Mrzljak et al., 1992). Along with dendritic development, the number of synaptic connections increases, starting in the primary sensory areas during the second trimester and proceeding toward higher-order areas. The “boom time” for cortical synaptogenesis in the primary sensory areas extends over the third trimester and the first three postnatal months, resulting in a sixfold increase in synaptic density (Huttenlocher and Dabholkar, 1997). Of intracortical connections in the visual cortex, the first to form are the vertical intracolumnar connections around the 26–29th GW. Horizontal connections follow with intercolumnar projections within layers IVB/V forming around 37th GW and long-range horizontal connections within layer II/III only after 16 postnatal weeks (Burkhalter et al., 1993). These changes in cortical neural structures are also observable *in vivo* with DTI of the gray matter, where both the mean diffusivity (reflecting an increase in neurite number, cellular complexity, and synapse formation), and fractional anisotropy decline (reflecting an increase in dendritic elongation and branching orthogonal to cortical columns) (Ball et al., 2013). After the third postnatal month, the synaptic density in primary sensory areas decreases gradually, reaching adult levels around puberty (Huttenlocher and Dabholkar, 1997). Some studies on monkeys suggest that the most rapid phase of synapse elimination may occur as late as puberty (Bourgeois and Rakic, 1993).

Marked changes in several neurotransmitter systems also take place around term. One example is the effect of gamma-aminobutyric acid (GABA) on the post-synaptic neuron. In the adult brain, GABA is an inhibitory neurotransmitter. At early stages of development, however, GABA_A_ receptor activation leads to depolarization (i.e., synaptic excitation) of the post-synaptic neuron due to high intracellular Cl^−^ concentration (Ben-Ari et al., 2004). The change from GABAergic excitation to inhibition in humans likely takes place around or shortly after term (Dzhala et al., 2005).

Evaluating the effects of these changes in the intracortical wiring and “chemistry” of cortical neural processing *in vivo* is not straight forward, but some inferences can be made from careful examination of neurophysiological data. The deflections following the adult SEF_MN_20/SEP_MN_20 or neonatal sef_MN_30/sep_MN_30 are considered to represent a “higher” level of information processing either within local neural circuits of the primary cortical area or in higher-order cortical areas. In a developmental SEP study, Laget and coworkers (Laget et al., 1976) describe substantial changes in the early sep_MN_ sequence as a function of age. In awake term-age newborns, the early cortical sep_MN_ usually consisted of a wide initial surface negative potential in the parietal area, peaking at around 40 ms and lasting up to 100 ms. Already during the first postnatal month, a notch directed toward the baseline divided this wide neonatal response into two distinct peaks, the first of which peaked at 30 ms. The notch then grew in amplitude with increasing age and crossed the baseline at around 3–4 months of age. The overall sep_MN_ morphology attained an adult-like form by age 3 years (Laget et al., 1976). However, as only four recording electrodes were used and, consequently, no source modeling was applicable, it is difficult to infer whether the described changes represent the development of local or larger-scale processing.

Modeling the neural sources underlying the early cortical somatosensory responses in MEG has revealed fundamental, qualitative differences in the cortical activity pattern of neonates compared with school-age children or adults. In adults, regardless of the method of somatosensory stimulation, the hallmark of the early SEF in central contralateral areas is a quick transition from the initial, anteriorly pointing dipolar source (i.e., SEF_MN_20/SEF_T_30, Figure 2) to a more prominent posteriorly pointing source (i.e., SEF_MN_35/SEF_T_50, Figure 2). In neonates, no such posteriorly pointing source has been detected at all. Instead, in newborns, the activity of the initial anteriorly pointing source continues over the first 100 ms after both median nerve and tactile stimulation. This difference between adults and newborns holds with different interstimulus intervals (ISI) as well as different vigilance states (particularly also when adults are examined during sleep) (Nevalainen et al., 2008; Pihko et al., 2009). Thus, the neural populations generating the posteriorly pointing neural current source in adult SI are not similarly activated in neonates.

Some previous neonatal SEP studies seemingly disagree with the MEG data by reporting an “adult-like” initial parietal negativity followed by a positivity in the same area with only slightly prolonged latencies (Willis et al., 1984; Laureau et al., 1988; George and Taylor, 1991). This may, however, be an artificial effect of the highpass filter setting applied in these studies (see Pihko and Lauronen, 2004). Others using a lower highpass cutoff value showed a clearly distinct morphology of early neonatal SEPs compared with those of adults (Desmedt and Manil, 1970; Hrbek et al., 1973; Laget et al., 1976; Karniski, 1992; Karniski et al., 1992). Part of the differences between the SEF and SEP data also naturally arises from the different sources preferentially detected by the two methods: tangential in MEG and both tangential and radial in EEG/SEP.

The exact reason for the lack of a posteriorly pointing SI SEF component in newborns can only be speculated, since no general agreement exists on the cellular-level generation mechanism, even in adults (see, e.g., Huttunen, 1997). Whereas the earliest cortical response (SEF_MN_20/SEF_T_30 and SEP_MN_20) is generally agreed to represent thalamo-cortical excitation of pyramidal cells in area 3b of SI (Allison et al., 1989a), the mechanism underlying SEF_MN_35/SEF_T_50 is not as straight forward. During the SEF_MN_35/SEF_T_50, the intracellular current flow is directed from superficial to deeper cortical layers (i.e., opposite to that of SEF_MN_20/SEF_T_30). Such intracellular current could be generated by either inhibition in deeper cortical layers (Huttunen and Hömberg, 1991; Wikström et al., 1996; Restuccia et al., 2002; Huttunen et al., 2008), or by excitation of the pyramidal cell apical dendrites in layers I/II (Allison et al., 1989a). In area 1 of monkeys, both mechanisms seem important in the generation of a similar “superficial-to-deep” intracellular current dipole following the initial “deep-to-superficial” dipole (Gardner and Costanzo, 1980; Gardner et al., 1984; Kulics and Cauller, 1986; Cauller and Kulics, 1991; Nicholson Peterson et al., 1995). In newborns, the absence of a posteriorly pointing source could reflect a lack of functional cortico-cortical connectivity necessary for mediating the response, since many of these connections are established postnatally (Burkhalter et al., 1993; Kostovic and Jovanov-Miloševic, 2006). Alternatively, the possibly still immature GABAergic inhibition could result in absence of the posteriorly pointing SI SEF, since such response is also absent in patients with Angelman syndrome, a disorder caused by a deletion in the GABA_A_ receptor subunit gene (Egawa et al., 2008). Furthermore, the long-duration of the initial neonatal response might reflect prolonged excitation in the proximal parts of apical dendrites of the pyramidal cells in area 3b, due to, e.g., slow kinetics of intrinsic membrane conductances (Kim et al., 1995; Moody and Bosma, 2005) and immature neurotransmitter receptors.

**Figure 4:**
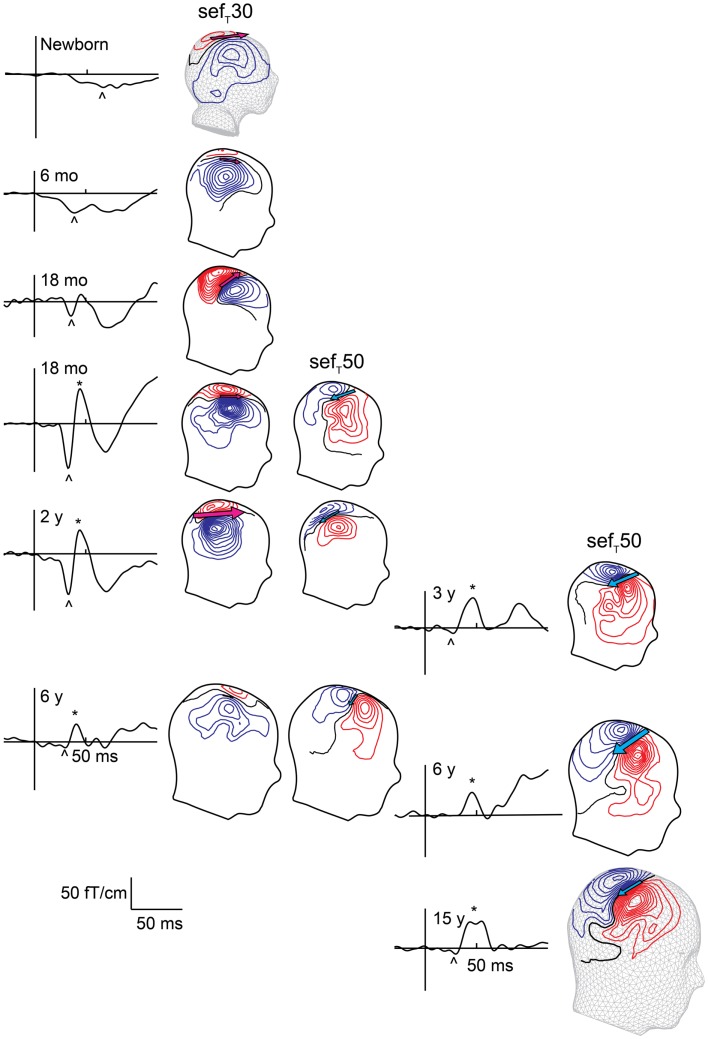
**Evolution of early sef_T_ responses with age**. Left column shows responses measured during sleep, and right column shows responses in awake subjects. The SEF waveform from one gradiometer channel shows the responses at different ages. The sef_T_30 is marked with an arrowhead and sef_T_50 with an asterisk. sef_T_50 appears around 1–2 years with inter-individual variation in timing and prominence. With age the amplitude of sef_T_30 decreases while that of the sef_T_50 increases. The magnetic contours – reflected on a head surface – reveal the different current orientations (indicated by the pink and blue arrows) underlying the sef_T_30 and sef_T_50: during the sef_T_30 the underlying current points anteriorly, and during the sef_T_50 posteriorly.

Though the exact mechanism for the lack of a posteriorly pointing SI SEF component in newborns remains unclear, it clearly reflects immaturity of somatosensory cortical processing. Consequently, emergence of the posteriorly pointing SI SEF likely reflects maturation of the functional somatosensory network. The transition from the neonatal SI response to the adult-like response occurs gradually during the first couple of years of life (Figure 4, Pihko et al., 2009). By 6 months of age, the originally wide U-shaped neonatal tactile SEF-response has turned into a W-shaped response with an emerging notch (Gondo et al., 2001; Pihko et al., 2009). This notch gradually grows in amplitude and crosses the baseline at around age 2 years, when the field pattern, location and direction of the underling currents begin to resemble those of the adult SEF_MN_35/SEF_T_50. After 2 years of age, the morphology of the responses gradually turns into the mature form with the posteriorly pointing source becoming more and the anteriorly pointing less prominent (Figure 4, Pihko et al., 2009). In school-age children and adolescents, the SEF-response sequence (i.e., anteriorly pointing dipolar source followed by a prominent posteriorly pointing source), is very similar to that of adults (Lauronen et al., 1997; Lauronen, 2001; Xiang et al., 2003; Bast et al., 2007; Nevalainen et al., 2012a). In general, the developmental pattern observed with MEG has a lot in common with the SEP morphology development described by Laget et al. (1976). However, the developmental changes occur at a younger age in SEPs than SEFs, for which there are several possible explanations. First, the SEP study used median nerve stimulation, whereas the developmental SEF study applied tactile stimulation of the index finger. Second, the SEP recordings were performed in awake infants and children, whereas SEFs were recorded during sleep. Third, neural activity in gyri may have a greater contribution to the change in SEPs, whereas MEG mainly reflects activity within sulci. For example, the notch in Laget et al.’s (1976) data may represent activity in the crown of the postcentral gyrus (area 1), which could go unnoticed in MEG. A combined multielectrode EEG–MEG study would clearly provide invaluable information about the differences and similarities in SEP and SEF development and clarify the underlying developmental phenomena.

### Integration of somatosensory information in large-scale cortical networks

Relatively little is known about the development of long cortico-cortical connections and the functionality of large-scale neural networks in the prenatal and neonatal period. In humans, callosal unmyelinated fibers are detectable in DTI around the 28th GW (Hüppi et al., 1998a). Postmortem anatomical studies show that after the 35th GW, the long cortico-corticals (e.g., callosal fibers) grow into the cortical plate (Kostovic and Jovanov-Miloševic, 2006). In mice, the region- and layer-specific targeting of callosal projections in the contralateral SI is dependent on electrical excitation and synaptic output of the callosal neurons (Wang et al., 2007). Furthermore, in rhesus monkeys, callosal axons are first overproduced, being three to four times more numerous in newborn than adult monkeys, and their refinement takes place postnatally through callosal axon elimination (LaMantia and Rakic, 1990). Maturation of the callosal connections in humans continues years after birth, as indicated by DTI anisotropy of the corpus callosum, which increases between childhood (7–11 years) and adolescence (15–17 years) (Koerte et al., 2009). Specific knowledge about the development of non-callosal, long cortico-cortical connections is sparse. In general, cortical projection neurons initially have relatively widespread distributions, which become more restricted during development through elimination of functionally inappropriate axon segments and branches (O’Leary et al., 2007).

Recent fMRI data suggest that several resting-state functional networks, including a network encompassing bilateral sensorimotor regions, are present at term-equivalent age (Fransson et al., 2007, 2009; Lin et al., 2008). The neonatal resting-state networks are, however, relatively restricted to homotopic counterparts in the two hemispheres with strong interhemispheric but limited intrahemispheric connectivity, whereas in adults, highly integrated interhemispheric and intrahemispheric connections between disparate regions exist (e.g., Biswal et al., 2010). In general, an increase in strength, complexity, and regional variability of networks are the hallmarks of resting-state fMRI across all periods of development that have been investigated (Lin et al., 2008; Fair et al., 2009; Gao et al., 2009; Supekar et al., 2009; Smyser et al., 2010). Accordingly, the interhemispheric connectivity between the left and right sensorimotor cortices increases both during the preterm period (Doria et al., 2010; Smyser et al., 2010) as well as between birth and age 2 years (Lin et al., 2008; however, see also Liu et al., 2008). Differences in the architecture of the resting-state networks between newborns and adults have also been demonstrated with a graph-theoretical analysis approach to fMRI data. In the adult brain, cortical hubs (brain areas with a disproportionately high degree of functional connectivity, suggesting an important role in the control of information flow) and their related cortical networks are mainly located in higher-order association areas such as posterior cingulate, lateral temporal, lateral parietal, and medial/lateral prefrontal cortices (Achard et al., 2006; Buckner et al., 2009). In contrast, in newborns, cortical hubs and their associated cortical networks are mainly found in primary sensory and motor brain regions (Fransson et al., 2011). However, the resting-state (or background) neural activity during early development and adulthood are different both in terms of generative mechanisms and function. Consequently, the results of developmental resting-state studies using indirect measures of neural activity (such as hemodynamic changes in fMRI) should be interpreted conservatively (Colonnese and Khazipov, 2012). In adults resting-state fMRI activity is thought to reflect the slow modulation of ongoing oscillatory activity generated by dense cortico-cortical networks. During early development (particularly during prematurity), however, the source of the slow fluctuations in resting-state fMRI may reflect alternating periods of electrical silence and bursts generated in sensory networks, driven by spontaneous activity in the periphery that interacts with the thalamo-cortical networks (Colonnese and Khazipov, 2012).

Altogether, studies of resting-state networks and brain anatomy suggest that at the time of full-term birth, the large-scale brain networks are immature and their connectivity patterns restricted. However, both fMRI and MEG studies in newborns have demonstrated ipsilateral SI responses, and MEG studies have revealed bilateral SII responses at term-age.

In fMRI, unilateral passive finger extension–flexion movements elicited BOLD (blood-oxygen-level dependent) responses in contra- and ipsilateral SI without significant differences between the hemispheres. This was interpreted as immature lateralization of somatosensory processing in newborns (Erberich et al., 2006; Heep et al., 2009). MEG responses from ipsilateral SI are detectable only in a minority of healthy newborns, however, and have longer latencies (approximately 20-100 ms) than the contralateral responses (Nevalainen et al., 2008). This suggests that the ipsilateral SEFs are unlikely to represent cortical activation via direct thalamo-cortical pathways, but could be generated through callosal connections that can be relatively abundant at term-age (LaMantia and Rakic, 1990). The millisecond scale latency difference between the contra- and ipsilateral MEG SI activations in neonates would go unnoticed in fMRI, likely explaining the lack of difference between signals at contra- and ipsilateral SI reported by Erberich et al. (2006). Interestingly, at 3 months of age follow-up, the BOLD fMRI signal changes already exhibit an adult-like contralateral SI activation, which persisted at 6 and 9 months (Erberich et al., 2006; Heep et al., 2009). No MEG or EEG reports exist on ipsilateral SI response development in early childhood after the neonatal period.

In adults, unilateral tactile stimulation elicits ipsilateral positive BOLD signals in the posterior parts of SI, probably area 2, in agreement with the bilateral representation of digits in area 2 in monkeys (Fabri et al., 1999; Polonara et al., 1999; Iwamura et al., 2002). With MEG, activation of ipsilateral SI in adult subjects has been detected in some studies (Korvenoja et al., 1995; Kanno et al., 2003; Hadoush et al., 2010; Pihko et al., 2010). Intracranial recordings confirm the presence of ipsilateral SEPs in some epilepsy and tumor patients. In contrast to contralateral responses, they originate only from Brodmann areas 1 and 2, have longer latencies, smaller amplitudes, and no initial surface negativity or phase reversal across the central sulcus (Allison et al., 1989b; Noachtar et al., 1997). The difference in sensitivity to the direction of underlying currents (mostly radial from area 1) and synchronization of activity could explain why ipsilateral responses from areas 1 and 2 are detected better with fMRI than MEG. Another explanation for the rarely reported ipsilateral SI responses in adults may involve transcallosal inhibition of ipsilateral area 3b after unilateral somatosensory stimulation (Hlushchuk and Hari, 2006). In the motor system, transcallosal inhibition is absent in infancy and early childhood (Müller et al., 1997; Heinen et al., 1998). The presence of ipsilateral SI responses could reflect lack of transcallosal inhibition in the somatosensory system in early childhood.

Knowledge of somatosensory processing beyond SI was limited before the era of MEG and modern neuroimaging. Since then, these methods have shown that, in the mature somatosensory system, information processing takes place in a wide network, including at least frontoparietal operculum (e.g., the bilateral SII), posterior parietal cortex, and mesial paracentral lobule (see, e.g., Hari and Forss, 1999 for MEG and Disbrow et al., 2000 for fMRI).

**Figure 5:**
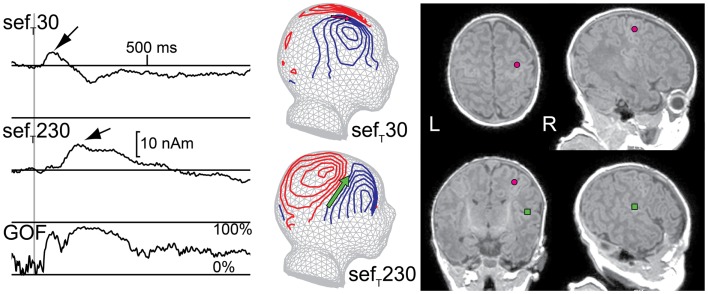
**Responses from primary (SI) and secondary (SII) somatosensory areas in a newborn**. Left: multidipole model showing SI and SII source waveforms for the right hemisphere of one newborn. Middle: magnetic field patterns during M60 (SI) and M200 (SII) peaks reflected on a spherical surface and projected onto a schematic newborn head. Right: SI (pink circle) and SII (green square) ECD locations on the newborn’s axial, coronal, and sagittal magnetic resonance images (MRIs). (GOF = goodness of fit)

Neonatal MEG studies have demonstrated that, after tactile and median nerve stimulation, a prominent deflection in the contralateral hemisphere peaks at about 230 ms (sef_MN_230/sef_T_230; Figure 5). The generator source underlying this deflection has been localized to the parietal operculum and thus sef_T_230 most likely represents activity in SII (Pihko et al., 2005; Nevalainen et al., 2008, 2012b). This indicates that the connections to and the neurons in SII are sufficiently developed to produce a detectable SEF response at full-term-age, although with a longer peak latency (approximately 230 ms) than in adulthood (approximately 100 ms; e.g., Hari and Forss, 1999). The neonatal SII response does have some similar characteristics with the mature SII response: it is often detectable not only contralaterally but also ipsilaterally and is diminished with the shortening of the ISI (Nevalainen et al., 2008). Interestingly though, the neonatal SII response is particularly prominent in quiet sleep (Pihko et al., 2004; Nevalainen et al., 2008), whereas in adults it is diminished or even vanishes in non-REM sleep (Kitamura et al., 1996; Kakigi et al., 2003; our own unpublished observation). Thus, from an ontogenetic point of view, SII activation in quiet sleep in newborns may have a function in the maturation of the somatosensory neural network. No reports exist about the development of SII responses in early childhood after the neonatal period.

In neonatal SEP studies, several investigators consistently detected a peak at a latency of around 230 ms (Desmedt and Manil, 1970; Hrbek et al., 1973; Laget et al., 1976; Karniski, 1992; Pihko and Lauronen, 2004; Pihko et al., 2004), with a positivity at the vertex (Desmedt and Manil, 1970; Pihko and Lauronen, 2004; Pihko et al., 2004). Although this SEP response was usually recorded with only a few electrodes, and hence no source modeling was applicable, in retrospect with knowledge from MEG studies, it can be interpreted to represent activity from SII. SEP studies on the evolution of this response with age are also lacking.

## Early Brain Insults and Somatosensory System Development

The “age-specific” nervous system of children affects the way in which neural dysfunction presents itself in childhood. First, the lesion types themselves depend on age, since periods of specific neurodevelopmental events are also periods of specific vulnerability. For example, asphyxia in preterm infants preferentially affects periventricular regions, whereas in term infants, cortical regions, thalamus, basal ganglia, and brainstem are more often affected. Second, whereas in adults neurological dysfunction causes specific and localized signs (e.g., hemiplegia in case of stroke in the medial cerebral artery area), in young infants it may present as generalized and non-specific symptoms (e.g., a preterm infant with a unilateral intraventricular hemorrhage (IVH) may present with generalized hypotonia or hypertonia, hypokinesia, or hyperexcitability) (Hadders-Algra, 2004). Furthermore, due to ongoing brain development, the long-term outcome after brain insults occurring prenatally or in early infancy is very different from outcome after an insult occurring in adulthood. The capacity for plastic reorganization after insults is much greater in the developing, immature brain. On the other hand, the mature patterns for information processing that need to be regained after the insult never existed in the immature brain in the first place, and the insult may instead compromise normal developmental processes (Kolb, 1999). The outcome after an early insult is also difficult to predict, since neurodevelopmental changes can induce a disappearance of observed symptoms present at an earlier age. Alternatively, functional deficits may only be recognized with increasing age because of the age-related increase in the complexity of neural functions (Hadders-Algra, 2002). The pathogenic mechanisms leading to the variable neurological deficits after early brain insults are also poorly understood, as *in vivo* studies of the functional development of the human brain have only been enabled recently by non-invasive investigation methods. In pediatric patient populations, MEG studies may, thus, be aimed at resolving questions concerning pathophysiological processes or to find neurophysiological biomarkers for predicting outcome in risk groups. Examples of both approaches are provided in the following sections.

### SEFs in predicting neurodevelopmental outcome in preterm infants

Although the survival of very preterm infants has increased significantly during recent decades (Vohr et al., 2005), many of these infants still develop with neurological impairments. Neonatal neurological examination is challenged by the non-specificity of signs of neural dysfunction (discussed above) and, consequently, complementary biomarkers for adverse outcome have been sought from neuroimaging and neurophysiology. As the period of active dendritic development and synapse formation is likely to offer better possibilities for rehabilitation than later periods (Kolb, 1999), interventions need to be started at an early age (note, however, the suggested reasons for restricting interventions before term-age: De Graaf-Peters and Hadders-Algra, 2006). Consequently, early prediction of outcome is essential for rehabilitation resources to be offered to those most in need.

Classical risk factors for adverse neurological outcome, including cystic periventricular leukomalacia (PVL) and grade III–IV IVH picked up by cranial ultrasound (Neil and Inder, 2004), are seen ever more rarely, and preterm infants with normal cranial ultrasound may have adverse outcomes (Laptook et al., 2005). At present, the type of pathology suggested to account for most of the neurological problems of preterm infants is diffuse white matter injury (WMI) (Khwaja and Volpe, 2008). WMI is further associated with impaired cerebral cortical development (Inder et al., 1999) and is also likely to lead to impaired development of cortico-cortical connectivity (Mathur and Inder, 2009). In preterm infants, moderate to severe white matter abnormalities in MRI at term-age are associated with severe cognitive and motor dysfunction at 2 years of age (Woodward et al., 2006) and cognitive, language and executive function impairment at 4 and 6 years of age (Woodward et al., 2012).

Furthermore, defects in the microstructural development of the cortex, reflected in DTI as higher mean diffusivity and fractional anisotropy in gray matter in preterm vs. term infants at term-equivalent age, may underlie adverse neurodevelopmental outcome. A slower decline in mean diffusivity during the preterm period was associated with lower overall developmental scores in Griffiths Mental Development Scales at 2 years corrected age (Ball et al., 2013). Moreover, at term-equivalent age, fMRI data have demonstrated significant differences between the resting-state networks of preterm infants and term-born controls. Specifically, functional connectivity between thalamus and sensorimotor cortex was weaker in the preterm infants than in the full-term infants (Smyser et al., 2010).

Possible injury to the thalamo-cortical connections due to periventricular pathology has also motivated a wealth of SEP studies in preterm infants to assess the predictive value of abnormal (absent or delayed depending on the study) median nerve sep_MN_30 (usually referred to as N1 in the literature; Klimach and Cooke, 1988b; Willis et al., 1989; de Vries et al., 1992; Pierrat et al., 1997) and the earliest posterior tibial nerve SEP (referred to as P1) from SI for future cerebral palsy (CP) (White and Cooke, 1994; Pierrat et al., 1997; Pike and Marlow, 2000). Results have been, however, somewhat contradictory, with specificity, sensitivity, and positive and negative predictive values varying markedly between studies. At least part of this variation is probably explained by differences in patient inclusion criteria, methods of SEP assessment, and outcome measure as well as technical difficulties in reliably recording the responses, particularly in the youngest infants (Smit et al., 2000).

A recent MEG study has expanded the somatosensory response evaluation from SI to SII, thus including cortico-cortical processing assessment. SEFs were recorded at term-age from 39 extremely preterm infants (born <28 GW) whose neurodevelopmental outcome was assessed at 2 years corrected age (Rahkonen et al., 2013). MEG data showed that, while SI responses were present in all the preterm infants, the SII response was absent uni- or bilaterally in a third of them. At follow-up, those infants with SII responses missing at term had worse neuromotor and overall developmental scores in the Griffiths Mental Developmental Scales assessment than the preterm infants with normal SII response present at term (Rahkonen et al., 2013). On the contrary, in this study, mild white matter abnormalities in MRI at term-age were not associated with adverse neurodevelopment (the study group did not include infants with moderate/severe WMI) (Rahkonen et al., 2013). It was speculated that the absence of SII responses may reflect not only damage to the connections within the sensorimotor networks but also more widespread disturbances in the development of cortico-cortical connectivity. Behaviorally, SII areas are considered to be involved in the integration of somatic inputs across large portions of the hand, sensorimotor integration, and bimanual coordination (see, e.g., Disbrow et al., 2000 for a discussion), motivating further SII response studies in preterm infants. Many such infants develop with minor neuromotor dysfunction and poor coordination (Hadders-Algra, 2002; Saigal and Doyle, 2008).

### MEG investigations of sensorimotor system reorganization after lesions to the developing brain – CP patients

Cerebral palsy, caused by an early lesion to the developing brain, is a persistent disorder of movement and posture often accompanied by various sensory deficits. When the early lesion is unilateral and severe enough, in some individuals the sensorimotor networks may develop into an unusual configuration. During normal development, the originally bilateral cortico-spinal motor innervation (Eyre et al., 2001; Eyre, 2003) is reduced to mainly contralateral connections during the first two postnatal years through elimination of most ipsilateral projections. However, after an early unilateral brain insult, the ipsilateral cortico-spinal projections can be, to a great extent, maintained. In such cases of hemiplegic CP, instead of the normal contralateral motor representation, transcranial magnetic stimulation (TMS) may demonstrate bilateral or completely ipsilateral motor representation of the plegic extremity depending on the timing, location, and extent of the lesion (Staudt et al., 2002, 2004, 2006; Eyre, 2007). The mechanisms for preservation of the ipsilateral cortico-spinal projections are thought to involve activity-dependent competition for spinal synaptic space (Eyre, 2007).

Contrary to the motor system, after both subcortical and cortico-subcortical early brain lesions, primary somatosensory representation of the affected hand generally remains in the contralateral (i.e., ipsilesional) hemisphere as demonstrated by MEG (Gerloff et al., 2006; Staudt et al., 2006; Wilke et al., 2009; Nevalainen et al., 2012a; Pihko et al., 2014), SEPs (Guzzetta et al., 2007), and fMRI (Wilke et al., 2009). Consequently, contralesionally organized motor representation results in the dissociation of somatosensory and motor cortical representations (Staudt et al., 2006; Guzzetta et al., 2007; Wilke et al., 2009). With limited interhemispheric and intrahemispheric reorganization capability, the somatosensory system seems to be particularly vulnerable to lesions extending to the neocortex (Wilke et al., 2009), especially those including areas SI, SII, and/or inferior parietal cortex (Juenger et al., 2011). When the cortex is spared, ascending thalamo-cortical tracts are able to bypass subcortical periventricular white matter lesions, as shown by DTI, and connect to their normal destinations in SI, even in patients with extensive subcortical lesions and ipsilateral motor representation (Staudt et al., 2006). Clinically, somatosensory abilities in such patients are relatively well preserved (Wilke et al., 2009), despite the fact that the fiber count in the thalamo-cortical somatosensory tract may be reduced (Thomas et al., 2005).

In CP patients with unilateral subcortical lesions and contralateral somatosensory representation, somatosensory MEG data reveal alterations in cortical somatosensory processing in both hemispheres (Nevalainen et al., 2012a). First, after median nerve stimulation, the normal activation sequence of SI (SEF_MN_20–SEF_MN_35) was disrupted in both hemispheres by an additional peak, SEF_MN_25, with posterior current orientation, preceding a delayed SEF_MN_35. Second, within SI, the cortical representations of contralateral digits II and V were located abnormally close to each other in both hemispheres. Unilateral spatial alterations in the hand representation area have been detected by fMRI, where the area activated by passive hand movement showed larger inter-individual spatial variability in the affected than unaffected hemisphere in patients with CP (Wilke et al., 2009). Third, compared with typically developing adolescents, in the CP patients, SEFs from ipsilateral SI in response to stimulation of the normal hand were more frequent. Thus, the ipsilateral responses in the CP patients do not suggest contralesional (i.e., ipsilateral) reorganization of the somatosensory representation from the affected hand (Nevalainen et al., 2012a). They may, however, reflect another form of unusual organization of the sensorimotor networks and/or lack of the usual ipsilateral area 3b inhibition (Hlushchuk and Hari, 2006). Deficient transcallosal inhibition between the motor cortices has been demonstrated with TMS in some (Heinen et al., 1999), though not all, diplegic CP patients (Koerte et al., 2011).

The dissociation of primary motor and somatosensory representations into different hemispheres in some individuals with CP offers a unique opportunity to study reactivity of sensorimotor oscillations separately from SI and the primary motor area (MI). (Usually, SI and MI locations in the post- and precentral gyri are in such close proximity to each other that separating sources originating from the two areas with certainty in MEG is difficult.) In three CP patients, Gerloff et al. (2006) observed using MEG the corticomuscular coherence to voluntary, paretic hand isometric contraction in the ipsilateral precentral gyrus at a location similar to where TMS evoked responses in the paretic hand muscles. This result suggests that the cortical signal coherent with electromyography during isometric contraction represents the driving volley from MI, and not sensory feedback processing within SI. In another study, in two CP patients with dissociation of motor and somatosensory representations, stimulation of the median nerve of the affected hand did not modulate the sensorimotor beta-band oscillations in either hemisphere, even though oscillations in alpha-band were modulated in both hemispheres (Pihko et al., 2014). These data suggest that the somatosensory afferent flow to the contralateral cortex was unable to influence the excitability of the motor cortex in the ipsilateral hemisphere controlling the affected hand.

Discoveries of the large-scale alterations in the wiring of the sensorimotor brain networks in people with CP clearly demonstrate the power of multimodal functional investigation of the developing brain. MEG is an essential tool in the brain development investigation toolbox, particularly when used in combination with other non-invasive methods, such as DTI, fMRI, and TMS. The puzzle of brain network organization after early insults of different scale, timing, and etiology, however, still remains only partly solved. The heterogeneity in this patient population calls for large multi-methodological studies to reveal the principles of vulnerability vs. plastic potential in brain organization after early brain insults.

## Limitations of MEG in Developmental Somatosensory Studies

Studying children – infants in particular – with MEG or most of the other new non-invasive brain research methods is not and will never become as straightforward as studying adults. First, up to a certain age, only passive paradigms are applicable. Moreover, as most of the new research methods require staying either relatively (e.g., MEG) or completely (e.g., fMRI) still, the youngest subjects can only be studied during sleep (or anesthesia), which requires a lot of time and patience and may alter the results. Furthermore, when studying brain function during sleep, the sleep stage needs to be carefully monitored since the background neural activity changes dramatically between different sleep stages, thus the possible effect of sleep stage on the phenomena of interest cannot be ignored (in fact, it would be rather surprising if sleep stage had no effect). To compare groups, the recordings need to be done at the same vigilance and/or sleep stage, which usually lengthens the recording times. In our experience, most of the failures in infant MEG measurements result from the infant not falling asleep within the reserved time slot.

**Figure 6:**
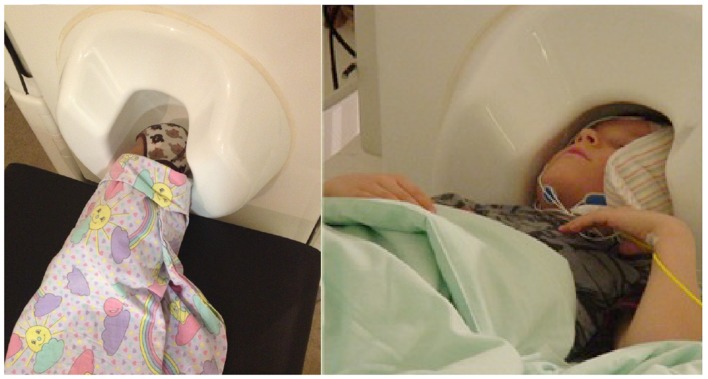
**Positioning infants and children in the MEG sensor array for somatosensory recordings**. Left: the head of a newborn can be positioned sideways in an adult-size MEG helmet so that one hemisphere is close to the sensors (occipital part of the helmet) and activity from that hemisphere can be recorded. Right: 6-year-old child sleeping with his head in an adult-size helmet. The head is positioned close to the right side of the helmet, the left hemisphere is further away from the sensors, and a small pillow fills the gap, restricting head movements.

In addition, we have faced challenges in head position measurement more often in measurements of infants than adults or older children. These problems are likely to arise from the disproportionately large size of the adult sensor helmet compared with the newborn’s head, resulting in a longer distance between some of the position indicator coils and the MEG sensors and, consequently, worse signal-to-noise ratio for the head position measurement. For reliable SEF recordings, the head of a newborn/infant needs to be close to the surface of the measuring helmet (Gaetz et al., 2008). With an adult-sized sensor array, this is possible only for one hemisphere at a time (Figure 6). The babySQUID, a special MEG instrument with infant-size but open headrest covering only part of one hemisphere, has the same problem (Okada et al., 2006; Papadelis et al., 2013). Thus, the number of possible paradigms in infants is reduced as, e.g., recording from both hemispheres simultaneously is not feasible. This doubles the measurement time when both ipsilateral and contralateral activity is of interest and largely prevents studies of interhemispheric interactions. Recently developed pediatric-sized measuring helmets have already proven successful in studying pre-school-age children (Johnson et al., 2010; Tesan et al., 2010) and are likely to considerably enhance the variability of possible paradigms for future MEG studies in the smallest subjects once still smaller helmets become commercially available (Edgar et al., 2012; see also Roberts et al., 2014 in this Research Topic).

Possible head movements during the measurement constitute another important issue in developmental MEG studies. Conducting recordings during sleep in the smallest subjects compensates for most of the movements, and continuous head position measurement likely deals with twitches during sleep (Taulu et al., 2004; Wehner et al., 2008). However, experience in applying continuous head position measurement in infants is at the moment sparse (Imada et al., 2006; Bosseler et al., 2013). In addition, artifacts in MEG produced by the electrical MN stimulation are greater in newborns, due to the proximity of the stimulation electrodes to the sensors. Therefore, non-electric tactile stimuli for somatosensory studies have turned out to be a good alternative in studies of infants.

As a clinical tool in our hospital, MEG is only used in a very limited number of patients, mainly those considered for epilepsy surgery to localize epileptic foci (e.g., Wilenius et al., 2013). MEG can also be used to localize the SI as part of pre-surgical functional mapping (e.g., Ochi and Otsubo, 2008; Tovar-Spinoza et al., 2008). In order for MEG to become a valuable tool for other purposes in the field of clinical neonatology or pediatrics, major developments in both machinery and analysis methods are still required. The devices should be mobile or located closer to neonatal units to allow studies of sick infants, and – in an optimal future scenario – the measurement helmet would be adjustable to the child’s head size. Furthermore, more automated analysis pipelines would greatly facilitate the possibility of using MEG as a tool to detect abnormality in neonatal brain activity.

## Conclusion and Future Prospects

In the last decade, MEG has provided a wealth of new information about normal and abnormal development of somatosensory cortical processing in early infancy, which would have been very hard to infer using other methods. Thus, a solid ground has been established to investigate the developing brain using MEG. With the somatosensory system, new information related to SII responses provides opportunities for studying higher cortical processing in neonates at risk for adverse outcome. Since neonatal care has significantly improved in the last decades, there is a growing need to detect the subtle disturbances that possibly cause problems in higher cortical functions but are evident clinically only later in life, e.g., during school years. MEG as a technique offers great possibilities for studying the pathophysiologic mechanisms underlying such subtle disturbances from a neuroscientific perspective.

## Author Contributions

Päivi Nevalainen had the main responsibility of writing the manuscript and preparing the figures. Leena Lauronen and Elina Pihko helped to design the outline and content of the manuscript and edited the manuscript and figures. Elina Pihko also prepared Figures 4 and 6.

## Conflict of Interest Statement

The authors declare that the research was conducted in the absence of any commercial or financial relationships that could be construed as a potential conflict of interest.

## Footnotes

^1^The nomenclature used in the SEF and SEP literature for the different response components is variable and easily confusing. It is based on a long tradition of SEP studies, where the components are named according to their latency and the direction of the potential difference in an electrode, e.g., N20 for a deflection at 20 ms with a negative value with respect to a reference electrode. SEFs are often named accordingly, e.g., N20m, with the *m* referring to “magnetic.” The nomenclature gets even more confusing with different stimulation methods and in recordings of infants/children, where latencies (and hence nomenclature) deviate from those of adults. For clarity, in this review, we will use SEF/SEP_MN_ and SEF/SEP_T_ for adult responses to median nerve and tactile stimuli, respectively, and sef/sep_MN_ and sef/sep_T_ for the respective responses of children. The number following the abbreviation of a response component refers to the latency of each response; e.g., SEF_MN_20 indicates adult SEF to median nerve stimulation peaking at 20 ms.

^2^Gestational weeks (GW), used in clinical practice to describe the length of pregnancy and fetus age, are traditionally calculated from the first day of the last menstruation (approximately 2 weeks before conception), but presently determined by ultrasound scans during pregnancy. For uniformity, we will use gestational weeks (rather than conceptional weeks calculated from the day of conception) throughout this review.
